# Morphological, thermal and mechanical properties of polypropylene foams via rotational molding

**DOI:** 10.1177/02624893211018825

**Published:** 2021-05-25

**Authors:** Yao Dou, Denis Rodrigue

**Affiliations:** Department of Chemical Engineering and CERMA, 98637 Université Laval, Quebec City, Quebec, Canada

**Keywords:** Polypropylene, rotomolding, chemical blowing agent, morphology, properties

## Abstract

In this work, polypropylene (PP) was foamed via rotational molding using a chemical blowing agent (CBA) based on azodicarbonamide over a range of concentration (0 to 0.5% wt.). The samples were then analyzed in terms of morphological, thermal and mechanical properties. The morphological analysis showed a continuous increase in the average cell size and cell density with increasing CBA content. Increasing the CBA content also led to lower foam density and thermal conductivity. Similarly, all the mechanical properties (tension, flexion and impact) were found to decrease with increasing CBA content. Finally, the efficiency of the rotomolding process was assessed by producing neat PP samples via compression molding. The results showed negligible differences between the rotomolded and compression molded properties at low deformation and rate of deformation indicating that optimal rotomolding conditions were selected.

## Introduction

Recently, scientific and industrial research increasingly focused on foamed thermoplastic materials, which are composed of a cellular core structure generated by the expansion of a blowing agent inside a thermoplastic matrix. This cellular structure allows the foams to be economically used in a wide variety of applications including automotive parts, protective equipment, building and construction, packaging industry and electromagnetic wave insulators.^1–3^ Additionally, foamed plastics present excellent cost to performance and strength to weight ratios compared with their unfoamed analogues because of their tunable weight reduction.^4,5^

Foamed polystyrene (PS) and polyethylene (PE) products have been commercially available for decades. However, their usefulness is limited due to their low heat deflection temperature.^6^ When PS foams are heated above their glass transition temperature (T_g_) around 100°C, they become soft and deform.^7^ Similarly, foamed PE is rarely used above 100°C because of its low melting point (110–130°C).^8^ Consequently, PS and PE foams are not suited for applications requiring elevated service temperature environments like contact with boiling water or sterilization processes. To overcome these PS and PE foams limitations at low cost, polypropylene (PP) foams attracted more and more attention recently. Firstly, the cost of PP has been 15–20% more competitive for the last decade compared to PE.^9^ Polypropylene, being a semi-crystalline polymer, enables to provide good flexibility and toughness, while having higher moduli and strengths. Secondly, PP offers better impact resistance than PS because PP is in a rubbery state at room temperature. Thirdly, due to its high melting point (160–175°C), high heat deflection temperature and service temperature is provided.^8^ Finally, PP has good chemical resistance (solvents, acids and bases).^10–12^ Therefore, benefitting from these advantages, PP foams have great potential for applications in the food and automotive industries.

So far, some studies have been conducted on PP foams manufactured by injection molding,^13–16^ compression molding^17–19^ and extrusion molding.^20,21^ For example, Ahmadi and Hornsby reported how the structure of PP foams was influenced by the injection molding processing conditions.^13^ The effect of injection speed, shot weight, mold temperature and melt temperature were investigated. The results showed that PP foams produced using a high injection rate, a low melt temperature and a high melt pressure exhibited a more uniform and finer cellular core structure. Mechraoui et al.^17^ produced PP foams by compression molding using different azodicarbonamide (ADC) concentrations (1.5, 2, 2.5 and 3% wt.) as a chemical blowing agent (CBA). As expected, the cell density, skin thickness and foam density decreased with increasing CBA content. The mechanical properties followed the foam density trend as less materials is available to support the applied loads.

In the last decades, the rotational molding technology gradually became one of the fastest-growing polymer processes in the plastic industries. This technology is a low-stress/shear process aiming at producing stress-free and seamless hollow plastic articles such as containers, tanks, toys, medical equipment and several similar products over a very wide range of dimensions and shapes.^22–29^ More recently, rotational molding was developed to process foamed parts. But very few works have been published on PP foams produced by rotational molding.^30–32^ Pop-Iliev and Park selected different PP grades in terms of melt flow rates (5.5 to 35 dg/min) to produce PP foams via rotational molding.^30^ It was observed that lower melt flow rates produced better fine-cell morphology and good expansion uniformity. Pop-Iliev et al.^31^ investigated how the narrow interval between the melting temperature of PP and the onset decomposition temperature of a CBA influenced the rotomolded PP foam structures. They concluded that desirable PP foam structures can only be achieved when PP sintering took place prior to the CBA decomposition to limit gas losses and the processing temperature kept low to limit cell coalescence. However, to the best of our knowledge, no studies reported the thermal and mechanical properties of PP foams manufactured by rotational molding.

The main objective of this work is to produce foamed and unfoamed rotomolded parts based on polypropylene. In particular, the effect of chemical blowing agent content is investigated to determine its relationship with foam density and cellular structure (cell size and cell density), as well as to determine its effect on the thermal (conductivity) and mechanical (tensile, flexural and impact) properties of PP foams. Finally, to confirm if optimal rotomolding conditions were selected, neat PP samples are also produced by compression molding to compare the properties obtained between both processing methods.

## Experimental

### Materials

The matrix selected was polypropylene (RMPP141 NATURAL) from PSD Rotoworx Pty Limited (Australia). This polymer has a melt flow index of 13 g/10 min (2.16 kg/230°C) and a density of 900 kg/m^3^. For foaming, an exothermic chemical blowing agent (CBA) based on activated azodicarbonamide was used: Celogen 754A (powder) from Chempoint (USA). Its peak decomposition temperature is 164°C as determined via DSC.

### Rotational molding

**Figure 1.**
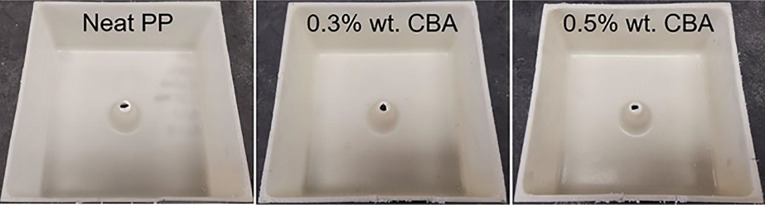
Typical examples of the rotomolded PP parts (cut samples).

A series of PP foams were prepared by using different CBA contents (0.1, 0.2, 0.3, 0.4 and 0.5% wt.) to compare with the unfoamed matrix (0% wt.). As the CBA must be thoroughly dispersed in the PP powder prior to charging the mold, all the materials were dry-blended in a high-speed mixer LAR-15LMB (Skyfood, USA) at 3320 rpm with fixed intervals of 1 min mixing time and 1 min cooling time repeated 5 times. A laboratory-scale biaxial rotomolding machine was used (MedKeff-Nye Roto-Lab model 22, Barberton, OH, USA). The parts were produced with a cubic aluminum mold (3.6 mm wall thickness and 19 cm internal side length). A demolding agent (Trasys 420, DuPont, Midland, MI, USA) was applied to the internal mold surface and a circular vent (10 mm diameter) was filled with glass wool to prevent powder losses. After preliminary trials, the optimum processing conditions were: a 3:4 speed ratio (major axis: minor axis), a heating time of 36 min with an electrically heated oven temperature of 270°C and a cooling time of 30 min with forced air convection. For each characterization, the samples were directly cut in the molded parts (Figure 1). Each sample was based on 660 g leading to final part thickness between 3.0 and 5.4 mm depending on the CBA concentration (expansion ratio).

### Compression molding

Compression molded PP parts were produced using 40 g of powder placed in a rectangular mold (110 × 110 × 3 mm^3^). An automatic Carver hydraulic press model Autoseries 3893 (Carver Inc., USA) at 190°C with a constant force of 2200 kg for 10 min was used before mold cooling to 60°C via water circulation before removing the pressure and demolding.

### Differential scanning calorimetry

Differential scanning calorimetry (DSC) studies were performed on a DSC-7 from Perkin-Elmer (USA) equipped with a thermal analysis controller TAC7/DX. About 15 mg was placed in sealed aluminum pans. The tests were performed between 50 and 220°C at 10°C/min under a flow of dry nitrogen (20 mL/min). The first heating cycle for PP was used to erase its thermal history and was not analyzed.

### Morphological characterization

The samples were cryogenically fractured (liquid nitrogen) and images of the cross-sections were taken by a scanning electron microscope (SEM) (FEI Inspect F50, USA). The average cell size (*D*) including standard deviation was determined by the ImageJ software (US National Institutes of Health, USA), while the cell density (*N_f_*), the number of cells per cubic centimeter of foam, was determined as^33^:


1 $$N_{f}\;=\;{\left.\left(\frac{n}{A}\right.\right)}^{3/2}$$

where *n* is the number of cells in a micrograph and *A* is the area of the micrograph in cm^2^.

### Density and hardness

The density was determined by cutting each sample into cubes. The dimensions were measured with a digital caliper (Mastercraft, Canada) with a resolution of 0.01 mm, while the weight was obtained from a MX-50 moisture analyzer (A&D, Tokyo, Japan), and compared with a gas (nitrogen) pycnometer Ultrapyc 1200e (Quantachrome Instruments, USA). The hardness (Shore A and Shore D) was measured by a PTC Instruments (USA) Model 306 L and Model 307 L following ASTM D2240, respectively. The results are the average and standard deviation of at least 5 repetitions.

### Thermal conductivity

The thermal conductivity (*k*) was determined by a home-made thermal conductivity analyzer based on ASTM E1225. The rotomolded parts were cut (50 × 50 mm^2^) and their thickness (*L* ± 0.01 mm) was measured using a digital caliper (Mastercraft, Canada). Each sample was placed between thin aluminum sheets (low thermal resistance) and the plate temperature (top = *T_h_*) and (bottom = *T_c_*) were fixed at 33°C and 13°C, respectively to give a 20°C difference (*▵T*) with a 23°C (room temperature) average. The temperatures were controlled by water cooled Pelletier plates (Model K20, Haake, Germany) and measured by thermistances (TC-720, TE-Technology, USA), while the equilibrium heat flux (*Q*) was obtained by a PHFS-01 heat flux sensor (Flux Teq LLC, USA). The data reported represent an average of three repetitions with standard deviations. The thermal conductivity was determined as:


2 $$k\;=\;\frac{Q\;L}{\Delta T}$$

### Mechanical properties

Tensile properties were determined according to ASTM D638 (type V) on an Instron (USA) 5565 with a 500 N load cell. The crosshead speed was 10 mm/min and the values (tensile modulus, tensile strength and elongation at break) are the average of six samples (±one standard deviation).

Flexural (three-point bending) tests according to ASTM D790 were done using a crosshead speed of 2 mm/min on an Instron (USA) 5565 with a 50 N load cell and a 60 mm span. Five samples (60 × 12.7 mm^2^) were used to report the average and standard deviation of the flexural modulus.

Charpy impact strength was obtained from a Tinius Olsen (USA) Impact 104. Ten specimens (60 × 12.7 mm^2^) were prepared according to ASTM D6110. The samples were “V” notched on an automatic sample notcher ASN 120 m (Dynisco, USA) at least 24 h before testing.

All the measurements were done at room temperature (23°C).

## Results and discussion

### Differential scanning calorimetry

**Figure 2.**
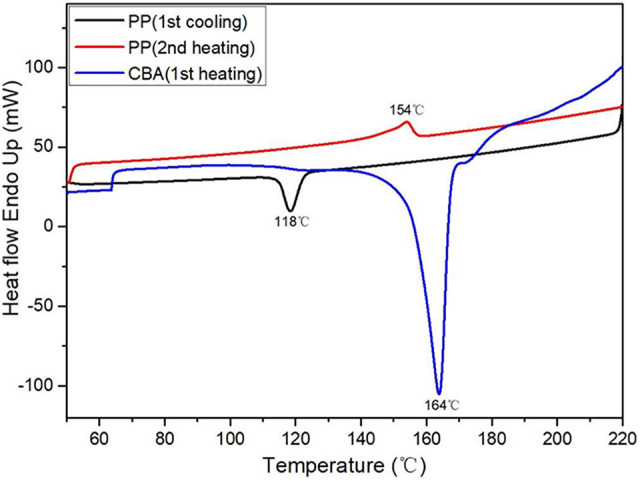
DSC thermograms of the PP and CBA used.

The DSC thermograms of PP and CBA are presented in Figure 2. The cooling and second heating cycle of PP show a peak melting temperature of 154°C and a crystallization peak temperature of 118°C. Figure 2 also shows that the CBA onset decomposition temperature is about 140°C with a peak decomposition temperature of 164°C. In this case, the onset of PP melting and CBA decomposition occurs at the same time leading to possible loss of gas. However, setting the oven temperature at 270°C for the rotomolding heating cycle enables a complete PP melt and CBA decomposition.

### Morphological characterization

**Figure 3.**
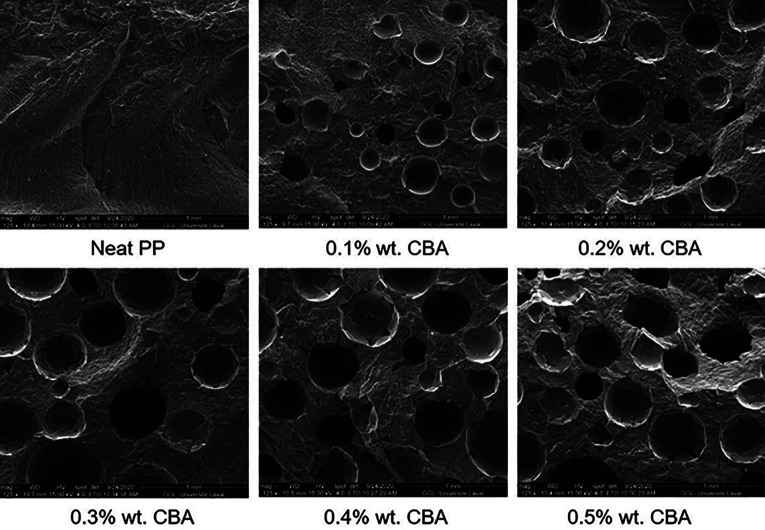
Typical morphologies of the rotomolded PP foams with different CBA contents.

SEM images for the foams with different CBA contents at low magnification (125×) are presented in Figure 3. From these images and their quantitative analysis, the average cell size and cell density are compared in Table 1. As expected, both parameters increase with increasing CBA content since more gas is available to blow the nucleated cells. For example, *D* increases from 0.212 to 0.331 mm, while *N_f_* increases from 7.0 × 10^3^ to 11.1 × 10^3^ cells/cm^3^ when the CBA content increases from 0.1% to 0.5% wt.

**Table 1.** Average cell size and cell density of PP foams.

CBA content (% wt.)	Average cell diameter (μm)	Cell density (10^3^/cm^3^)
0.1	212 ± 95	7.0
0.2	289 ± 117	7.6
0.3	298 ± 118	9.4
0.4	326 ± 74	9.8
0.5	331 ± 123	11.1

### Density and hardness

**Figure 4.**
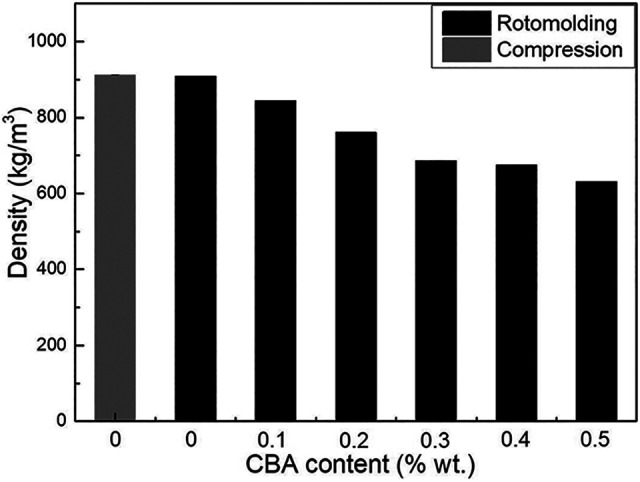
Density of PP and the foams as a function of CBA content.

**Figure 5.**
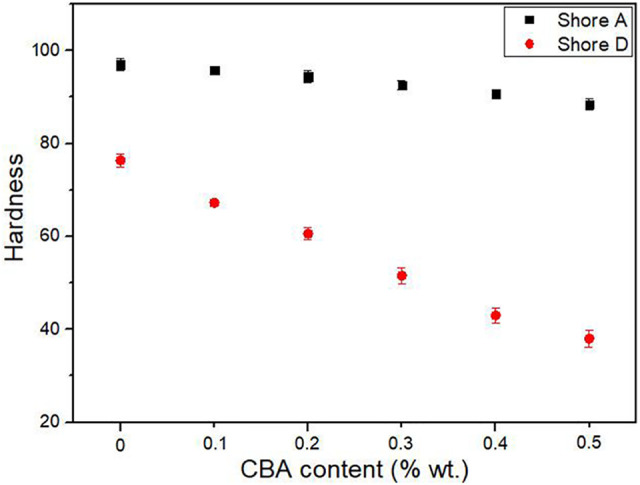
Hardness (Shore A and Shore D) of PP foams as a function of CBA content.

Figure 4 presents the density of the neat PP and the PP foams as a function of CBA content. Compared with the density of the rotomolded PP sample (0.908 g/cm^3^), the compression molded one has a slightly higher density with 0.912 g/cm^3^ resulting from the high pressure generating a more compact structure. With increasing CBA content up to 0.5% wt., the density decreases from 0.908 to 0.631 g/cm^3^ (31% reduction). Table 2 and Figure 5 show the hardness (Shore A and Shore D) of the neat PP and the PP foams as a function of CBA content, respectively. Based on the results of Table 2, similar hardness values of neat PP are obtained for the compression molded and the rotomolded samples. For the PP foams produced by rotomolding, an increase in the CBA content decreases the hardness values. For example, the Shore A decreases from 97.0 to 88.5 (8.5 points difference), while the Shore D decreases from 76.4 to 38.0 (38.4 points difference). Lower foam hardness is expected due to decreasing cell wall thickness (increasing cell size and cell density in Table 1) combined with a “softer” nature of the gas cells using more space inside the PP matrix.

**Table 2.** Hardness (Shore A and Shore D) of neat PP samples.

PP sample	Shore A	Shore D
Compression molding	97.5 ± 0.7	77.3 ± 0.9
Rotational molding	97.0 ± 1.3	76.4 ± 1.4

### Thermal conductivity

Table 3 presents the thermal conductivity results. The neat PP produced by rotomolding has a value of 145 mW/m.K which is similar to the sample produced by compression molding (149 mW/m.K). This result is in agreement with the reported thermal conductivity for PP (ranging from 0.10 to 0.22 W/m.K) in the literature.^34^ As expected, increasing the CBA content slightly decreases the thermal conductivity with the lowest value (104 mW/m.K) at 0.5% wt. CBA. This trend is similar as for foam density in Figure 4 indicating a direct correlation between both parameters.

**Table 3.** Thickness (*L*) and thermal conductivity (*k*) of the PP samples.

CBA content (% wt.)	*L* (mm)	*k* (mW/m.K)
0^c^	2.31	149 ± 5
0^r^	2.97	145 ± 6
0.1	3.44	143 ± 5
0.2	3.76	132 ± 6
0.3	4.20	118 ± 4
0.4	4.76	110 ± 5
0.5	5.37	104 ± 4

c: compression molded.

r: rotational molded.

### Flexural properties

**Figure 6.**
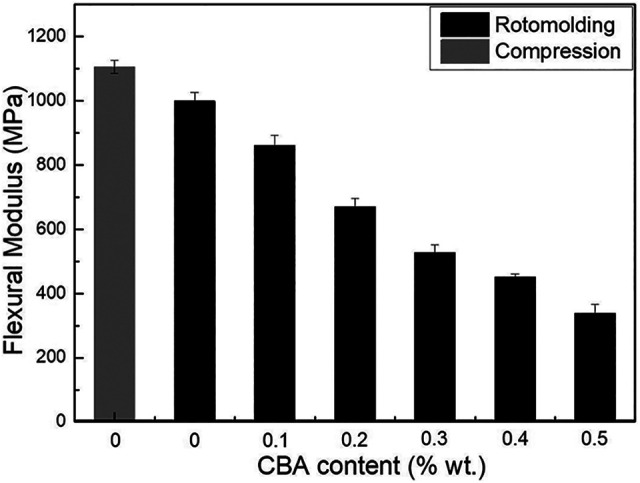
Flexural modulus of PP and the foams as a function of CBA content.

Figure 6 presents the flexural modulus of the neat PP and the foams. The value for the compression molded sample is 1105 ± 20 MPa which is slightly higher than the rotomolded one (998 ± 28 MPa). This might be related to the high pressure involved in compression molding leading to a better compaction (closer packing) and sintering of the particles leading to higher rigidity. Nevertheless, the values decrease with increasing CBA content. For example, the flexural modulus is 337 MPa at 0.5% wt. CBA which represents a 66% decrease. Lower values for foams are related to less material being available (decreasing density in Figure 4) to sustain the applied stress.^35^

### Tensile properties

**Figure 7.**
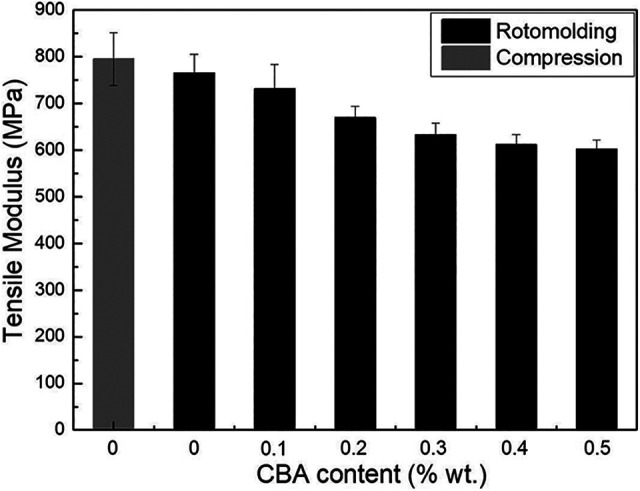
Tensile modulus of PP and the foams as a function of CBA content.

**Figure 8.**
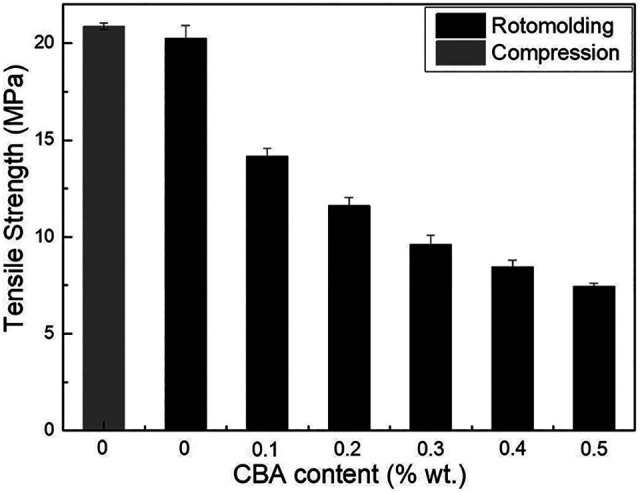
Tensile strength of PP and the foams as a function of CBA content.

**Figure 9.**
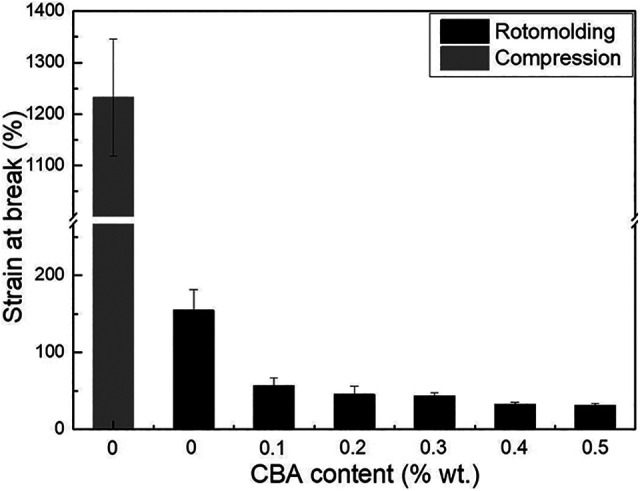
Tensile strain at break of PP and the foams as a function of CBA content.

For the tensile modulus (Figure 7), no significant difference between the compression molded (795 ± 56 MPa) and rotomolded (765 ± 41 MPa) samples was observed, while the same conclusion applies for the tensile strength (Figure 8) with values of 20.9 ± 0.2 MPa and 20.2 ± 0.7 MPa, respectively. However, the elongation at break (Figure 9) of the compression molded PP (1232 ± 113%) is much higher than that of the rotomolded one (155 ± 27%). This difference may be associated with the absence of pressure applied during rotomolding resulting in a looser molecular packing and a higher number of microvoids in the samples, which can be explained by the density difference between compression molding and rotomolding (Figure 4).^36^ These results indicate that differences between both processing methods (compression molding vs. rotomolding) are mainly important at higher deformation (elongation at break) compared to lower deformation (elastic modulus and maximum stress).

For the rotomolded foams, the tensile modulus (Figure 7) presents a similar trend as for the flexural modulus (Figure 6). The values decrease by 21% (from 765 to 602 MPa) with the addition of 0.5% CBA. Figure 8 also reveals that the tensile strength decreases with increasing CBA content, for the same reasons as for the flexural modulus. The tensile strength of the unfoamed matrix (20.2 MPa) decreased to 7.4 MPa (63% lower) at 0.5% wt. CBA. Finally, Figure 9 compares the elongation at break. Again, the values decrease with increasing CBA content and are all well below 100% (30% to 60%). All these results are in agreement with other studies reporting decreasing moduli, strengths and elongations at break with decreasing density.^37^

### Impact strength

**Figure 10.**
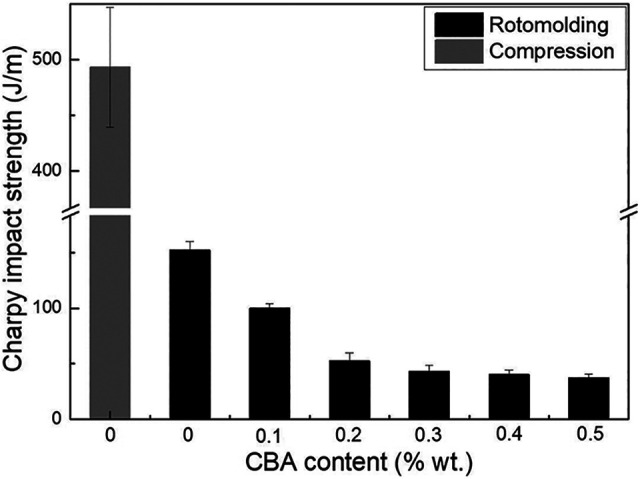
Impact strength of PP and the foams as a function of CBA content.

Impact strength is reported in Figure 10. In this case, a significant difference between the rotomolded (152 ± 8 J/m) and compression molded (493 ± 54 J/m) PP is observed because of a more compact structure of the latter. This indicates that another main difference between both processes occurs at high deformation rate.

The impact strength is also decreasing with higher CBA content. The values decrease from 152 J/m to 37 J/m (76% reduction) by adding 0.5% wt. The main origin of this trend is that the cells act as stress concentration points, and easier crack initiation and propagation occurs when larger cells are produced (Table 1) as the cell wall thickness decreases,^38^ which confirms our results.

### Final analysis

**Figure 11.**
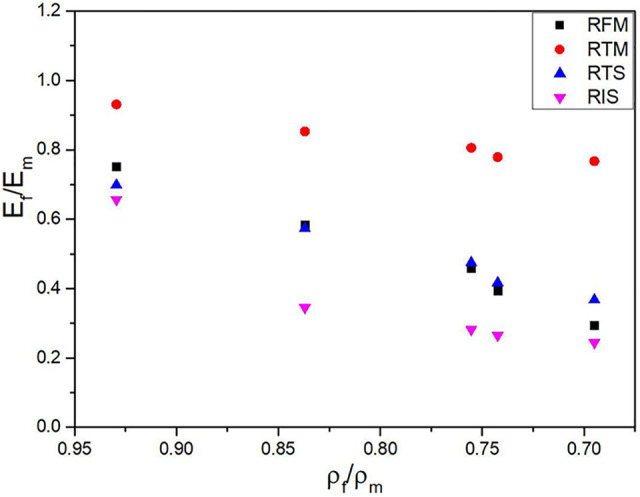
Relative mechanical properties (E_f_/E_m_) as a function of the relative density (ρ_f_/ρ_m_). RFM: relative flexural modulus, RTM: relative tensile modulus, RTS: relative tensile strength and RIS: relative impact strength.

To finalize our analysis, a comparison between the relative mechanical properties (property of the foam divided by the property of the matrix) and the relative density (density of the foam divided by the density of the matrix) is presented in Figure 11. These curves can be helpful to optimize a specific system (polymer, foaming agent, processing conditions, methods, etc.) since they account for both the mechanical response and density reduction. According to Figure 11, the rotomolded PP foams exhibit a continuously decreasing trend between both relative properties, indicating that no clear optimum was achieved for the range of conditions studied. Nevertheless, these foams would be better for applications in tension as their relative values are much higher than the other mechanical properties investigated.

## Conclusions

Polypropylene (PP) foams were successfully produced via rotational molding using a simple dry-blending of the chemical blowing agent (CBA) since both materials were in a powder form. The effect of CBA content (0-0.5% wt.) was evaluated on the morphological, mechanical and thermal properties. Finally, a comparison between compression molded and rotomolded neat PP parts was performed.

The results showed that tensile elongation at break and impact strength of compression molded samples were much better than rotomolded ones. Nevertheless, it is assumed that good processing conditions were used as the properties at low deformation and low rate of deformation were similar. This difference can be associated to the high pressure used in compression molding leading to better sintering and compaction of the final parts (slightly higher density).

From the morphological analysis, it was found that the average cell diameter and cell density increased with increasing CBA content. This was expected as more gas is produced improving cell nucleation and growth inside the PP matrix. As expected, a higher amount of porosity (lower density) led to lower hardness with CBA addition. Similarly, more void content improved the thermal insulation properties of PP foams as the thermal conductivity decreased from 149 mW/m.K to 104 mW/m.K (30% reduction) with only 0.5% wt. azodicarbonamide. Finally, due to the presence of a higher number of larger gas cells in the foams, less material is available to sustain the applied stresses with increasing CBA content leading to lower tensile and flexural moduli, as well as tensile strength and strain at break. A similar trend was observed for the impact strength because of larger cells acting as stress concentrators.

Nevertheless, more work is needed to optimize the processing of polymer foams, especially to completely understand the relations between all the parameters involved and their effect on the final structure and properties of rotomolded parts.
